# Warp-Knitted Spacer Fabric Reinforced Syntactic Foam: A Compression Modulus Meso-Mechanics Theoretical Model and Experimental Verification

**DOI:** 10.3390/polym12020286

**Published:** 2020-02-01

**Authors:** Chao Zhi, Mingjuan Du, Zhaoling Sun, Mengjie Wu, Xiaoyi He, Jiaguang Meng, Lingjie Yu

**Affiliations:** School of Textile science and engineering, Xi’an Polytechnic University, Xi’an 710048, China; zhichao@xpu.edu.cn (C.Z.); dumingjuan1022@163.com (M.D.); sunzhaoling1224@163.com (Z.S.); w18392165207@163.com (M.W.); hxy2475@163.com (X.H.)

**Keywords:** syntactic foam, warp-knitted spacer fabric, compression modulus, Meso-mechanics theoretical model

## Abstract

In this study, a new type ternary composite, called warp-knitted spacer fabric reinforced syntactic foam (WKSF-SF), with the advantages of high mechanical properties and a lower density, was proposed. Then, a meso-mechanics theoretical model based on the Eshelby–Mori–Tanaka equivalent inclusion method, average stress method and composite hybrid theory was established to predict the compression modulus of WKSF-SF. In order to verify the validity of this model, compression modulus values of theoretical simulations were compared with the quasi-static compression experiment results. The results showed that the addition of suitable WKSF produces at least 15% improvement in the compressive modulus of WKSF-SF compared with neat syntactic foam (NSF). Meanwhile, the theoretical model can effectively simulate the values and variation tendency of the compression modulus for different WKSF-SF samples, and is especially suitable for the samples with smaller wall thickness or a moderate volume fraction of microballoons (the deviations is less than 5%). The study of the meso-mechanical properties of WKSF-SF will help to increase understanding of the compression properties of this new type composite deeply. It is expected that WKSF-SF can be used in aerospace, marine, transportation, construction, and other fields.

## 1. Introduction

Syntactic foams are defined as closed pore foams which are formed by embedding hollow microballoons into matrix [1]. The hollow microballoons are usually made up of glass, phenolic, carbon or metal, while the matrixes are usually thermosetting resins [2].

Syntactic foams exhibit excellent properties, such as low density, high specific strength, high specific modulus and the possibility of tailoring mechanical properties by adjusting the types, as well as the volume fractions of microballoons [3]. Therefore, syntactic foams are being widely used in the fields of aerospace, marine, vehicles and architectural applications. Nevertheless, many researchers [4,5,6,7] have reported that the absolute strength of traditional syntactic foams is still insufficient, which severely limits further development of this composite.

Warp-knitted spacer fabric (WKSF) is a type of three-dimensional textile material, which consists of two separate surface layers joined together by spacer yarns [8]. As a rapidly developing textile material in recent years, WKSF shows excellent compression, impact resistance and cushioning properties. Meanwhile, adding WKSF into composite can effectively improve the interlaminar shear and compression strength of material in the thickness direction. Therefore, WKSF is especially adapted to the use of strengthening phases in composites [9,10,11].

On account of these advantages of WKSF, in our previous work, a new type of syntactic foam, namely WKSF-reinforced syntactic foam (WKSF-SF) was fabricated to improve the mechanical properties of syntactic foam, and the quasi-static compression properties of this material were studied [12]. The results indicated that the WKSF-SF made of preferable WKSF showed a higher compressive modulus and yield strength compared to neat syntactic foam (NSF). In addition, the compression performance of WKSF-SF can be changed by adjusting the parameters of WKSF and microballoons (such as the inclination angle of spacer yarns, surface layer structures, fineness of spacer yarns, contents or types of microballoons) according to the actual requirements. In view of the above, as a new type of ternary composite, WKSF-SF can solve the problem of insufficient absolute strength of traditional syntactic foam to a certain extent, and thus has great development potential.

However, current experimental researches for WKSF-SF are based on the macro perspective, so the reinforcement mechanism of WKSF on syntactic foam is not clear. Thus, it is difficult to achieve controllable preparation of WKSF-SF, which severely affects the further development of this new type of syntactic foam. Therefore, in order to understand the mechanical properties of WKSF-SF in depth, and then realize the controllable preparation of WKSF-SF, it is necessary to analyze the mechanical properties of WKSF-SF from the perspective of theoretical research and meso-mechanics. For the reasons above, establishing a meso-mechanics theoretical model for WKSF-SF, thus predicting the mechanical behavior of this new type of composite, is important.

In 1973, Mori and Tanaka published an important paper in which a method of calculating the average stress for the matrix of a composite [13], called the Eshelby–Mori–Tanaka equivalent inclusion method (referred to as the Mori–Tanaka method) was proposed. The Mori–Tanaka method is easy to operate and can directly give the display expression for the modulus of a composite. Therefore, this method was widely used to predict the mechanical properties of composites with ellipsoid-like inclusions [14].

Based on the above, this paper aimed to establish a theoretical compression modulus model for various WKSF-SF samples based on the Mori–Tanaka method, average stress method and composite hybrid theory. After that, the calculated compression modulus values are compared with experimental results to verify the accuracy of prediction models.

## 2. Materials and Methods

### 2.1. Materials and Sample Preparation

**Figure 1:**
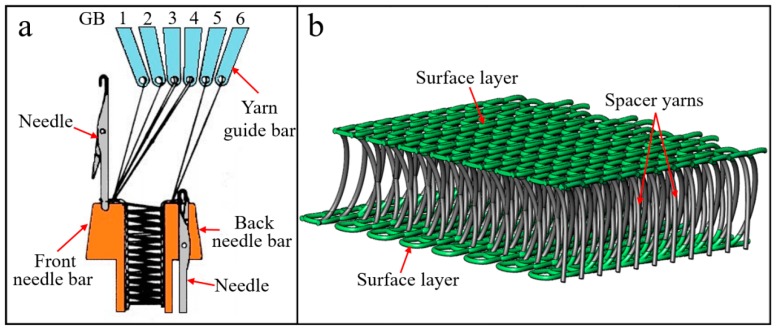
The (**a**) working principle of double-needle-bar warp-knitting machine and (**b**) schematic illustration of warp-knitted spacer fabric (WKSF).

**Figure 2:**
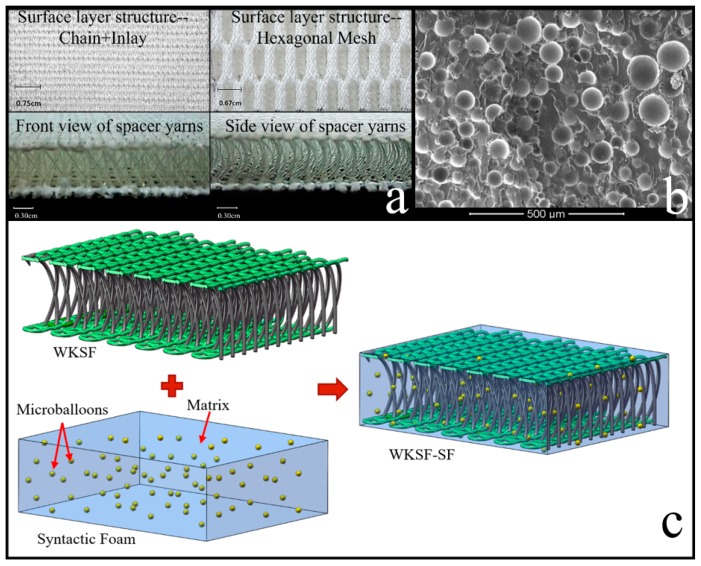
The structure of (**a**) WKSF, (**b**) microballoons and (**c**) schematic illustration of warp-knitted spacer fabric reinforced syntactic foam (WKSF-SF).

The materials (epoxy resin, hardener, microballoons and WKSF) and sample preparation process of the quasi-static compression test of SF-WKSF are mentioned in our previous paper [12,15], so they will not be covered again here. The specifications of all samples used in this research are summarized in Table 1. Meanwhile, the working principle of the double-needle-bar warp-knitting machine and schematic illustration of WKSF are shown in Figure 1. Figure 2a,b represents the structure of WKSF and microballoons, respectively, and Figure 2c represents the schematic illustration of WKSF-SF.

### 2.2. Specimen Nomenclature

The nomenclature of WKSF-SF samples can be illustrated by taking SWS60-30-W1 as an example. In this sample, SW denotes WKSF-SF, S60 represents the type of S60HS glass microballoons, 30 stands for the 30% volume fraction of the microballoons, and W1 denotes the type of WKSF added in the composite.

### 2.3. Quasi-Static Compression Test

Quasi-static compression tests in this research were refer to the ISO 844: 2014 (Rigid cellular plastics-Determination of Compression Properties) standard and carried out by a MTS 810 materials test system. Similarly, the compression test procedure can also be found in our previous work [12].

## 3. Modeling

**Figure 3:**
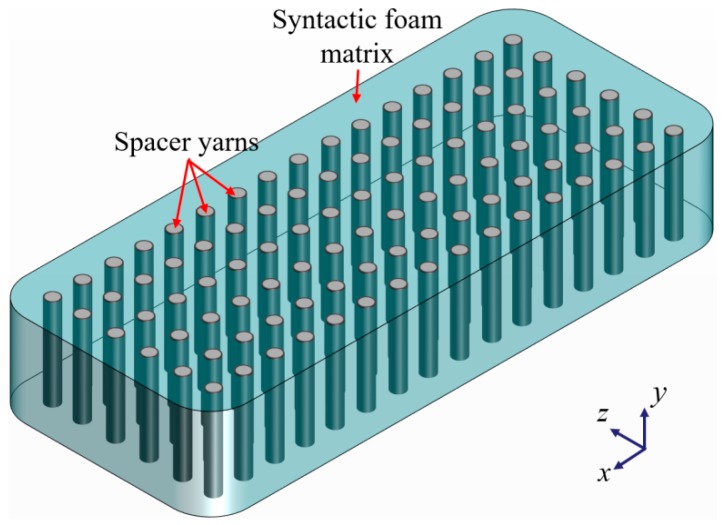
The equivalent model of WKSF-synthetic foam (SF).

When WKSF is subjected to the load through thickness direction, the spacer yarns are the primary load carrier, while the surface layers of WKSF have little contribution to carrying the compression load. Therefore, to simplify the model, the influence of surface layers of WKSF were not taken into account in the compression process. In addition, for the same purpose, the syntactic foam matrix (microballoons reinforced epoxy resin) is considered as homogenized in the modeling process. In conclusion, the WKSF-SF can be equivalent to a single-layer composite in which the spacer yarns are arranged in a single direction, as shown in Figure 3.

In the Mori–Tanaka method, by considering the mutual disturbance between the inclusion phases, also using the Eshelby equivalent inclusion theory and the average stress method [16], the equivalent elastic modulus ***L*** of composite can be obtained as follows

(1) $$L=L_{0}(I+V_{1}H{)}^{-1}$$

where *H* in the Equation (1) can be expressed as

(2) $$H={\left\{L_{0}+(L_{1}-L_{0})[V_{1}I+(1-V_{1})S]\right\}}^{-1}(L_{0}-L_{1})$$

where ***L***_0_ and ***L***_1_ are the elastic constants of the matrix phase and the reinforcement phase, respectively, ***I*** is the 4th order unit tensor, and ***S*** is the 4th order Eshelby tensor. ***V***_1_ is the volume fraction of the reinforcement phase.

For two-phase composites, G.J. Weng [17] gives a simplified form of the bulk modulus *K* and shear modulus *G*, based on the Mori–Tanaka method, as follows

(3) $$\frac{K}{K_{0}}=1+\frac{V_{1}(K_{1}-K_{0})}{(1-V_{1})\alpha (K_{1}-K_{0})+K_{0}}$$

(4) $$\frac{G}{G_{0}}=1+\frac{V_{1}(G_{1}-G_{0})}{(1-V_{1})\beta (G_{1}-G_{0})+G_{0}}$$

where *K*_1_ and *K*_0_ are the bulk modulus of the reinforcement phase and the matrix phase, respectively. Meanwhile, *G*_1_ and *G*_0_ are the corresponding shear moduli.

For the spherical particle reinforced composite (like syntactic foams), *α* and *β* in Equations (3) and (4) can be calculated by Equations (5) and (6), respectively [16]

(5) $$\alpha =\frac{1+\nu_{0}}{3(1-\nu_{0})}$$

(6) $$\beta =\frac{2(4-5\nu_{0})}{15(1-\nu_{0})}$$

where *ν*_0_ is the Poisson’s ratio of the matrix phase.

Substituting Equations (5) and (6) into (3) and (4), respectively, the bulk modulus *K*_NSF_ and shear modulus *G*_NSF_ for NSF can be expressed as follows

(7) $$K_{\mathrm{NSF}}=K_{e}[1+\frac{3V_{m}(1-v_{e})(K_{m}-K_{e})}{3K_{e}(1-v_{e})+(1+v_{e})(1-V_{m})(K_{m}-K_{e})}]$$

(8) $$G_{\mathrm{NSF}}=G_{e}[1+\frac{3V_{m}(1-v_{e})(G_{m}-G_{e})}{15G_{e}(1-v_{e})+2(4-5v_{e})(1-V_{m})(G_{m}-G_{e})}]$$

where *K*_e_, *G*_e_ and *ν*_e_ are the bulk modulus, shear modulus and Poisson’s ratio of matrix material, respectively, and each can be described as [18]

(9) $$K_{e}=\frac{E_{e}}{3(1-2v_{e})}$$

(10) $$G_{e}=\frac{E_{e}}{2(1+v_{e})}$$

where *E*_e_ is the elastic modulus of the matrix material.

In addition, in Equations (7) and (8), *K*_m_ and *G*_m_ represent the bulk modulus and shear modulus of the hollow microballoons, respectively. They can be expressed by using Equations (11) and (12) [19].

(11) $$K_{m}=\frac{2dE_{g}}{3r(1-v_{g})}$$

(12) $$G_{m}=\frac{5dE_{g}}{2r(7+5v_{g})}$$

where *E*_g_ and *ν*_g_ represent the elastic modulus and Poisson’s ratio of the microballoons material. Meanwhile, the *d* and *r* are the wall thickness and radius of the hollow microballoons, respectively.

Substituting the calculated *K*_e_, *G*_e_, *K*_m_, and *G*_m_ into Equations (7) and (8), the bulk modulus *K*_NSF_ and shear modulus *G*_NSF_ of NSF can be determined. And then, the elastic modulus *E*_NSF_ and Poisson’s ratio *ν*_NSF_ of NSF can be calculated from Equations (13) and (14) [18,19], respectively.

(13) $$E_{\mathrm{NSF}}=\frac{9K_{\mathrm{NSF}}G_{\mathrm{NSF}}}{3K_{\mathrm{NSF}}+G_{\mathrm{NSF}}}{\left(1-V_{v1}\right)}^{2}$$

(14) $$v_{\mathrm{NSF}}=\frac{3K_{\mathrm{NSF}}-2G_{\mathrm{NSF}}}{6K_{\mathrm{NSF}}+2G_{\mathrm{NSF}}}$$

where *V*_v1_ is the volume fraction of the voids in the composite. In order to consider the effect of voids on the elastic modulus of the composite, a correction term (1 − *V*_v1_)^2^ based on the square-law relationship [20] is added to Equation (15) to ensure the accuracy of the prediction result. According to the hybrid theory of composite, the final theoretical compression modulus model for the WKSF-SF can be expressed as

(15) $$E_{\mathrm{WKSF}-\mathrm{SF}}=\frac{9K_{\mathrm{NSF}}G_{\mathrm{NSF}}}{3K_{\mathrm{NSF}}+G_{\mathrm{NSF}}}{\left(1-V_{v1}\right)}^{2}V_{\mathrm{NSF}}+E_{\mathrm{SY}}V_{\mathrm{SY}}$$

where *E*_SY_ and *V*_SY_ are the elastic modulus and volume fraction of the spacer yarns in composite, respectively.

In order to verify the availability of this theoretical compression modulus model, compression modulus values of theoretical simulations need to be compared with the experimental results. Therefore, a total of 10 parameters in Equation (15) (*E*_e_, *ν*_e_, *E*_g_, *ν*_g_, *d*/*r*, *V*_v1_, *V*_m_, *E*_SY_, *V*_NSF_ and *V*_SY_) need to be determined.

Among them, *E*_e_ was obtained by a quasi-static compression test of the epoxy resin-cured product. The test instrument, materials (epoxy resin and curing agent), sample size, testing process and elastic modulus calculation process in quasi-static compression test were the same as the test of WKSF-SF, which are mentioned above. Then, the value of *E*_e_ was determined to be 1.01 GPa in this research. In addition, the Poisson’s ratio of epoxy resin *ν*_e_, the elastic modulus *E*_g_ and Poisson’s ratio *ν*_g_ of glass were determined as 0.3, 70 GPa and 0.23 [21], respectively. The values of *d*/*r* can be determined by the ratio of the inner and outer diameters of the microballoons. According to the manufacturer’s datasheet, the radius ratios of K1, S35 and S60HS were 0.98, 0.95 and 0.91, respectively. Based on the above data, the *d*/*r* values of hollow glass microballoons S60HS, S35 and K1 were 0.09, 0.05 and 0.02, respectively. Meanwhile, referring to our previous research [12], the value of *V*_v1_ was determined as 5% in this study. According to the addition amount of microballoons, the values of *V*_m_ for different WKSF-SF samples were 20%, 30% or 40%, as shown in Table 1. The value of *E*_SY_ was calculated based on the Polyethylene Terephthalate Polyester data sheet (provided by Goodfellow) and tensile testing, which was finally determined as 3.39 GPa. The *V*_NSF_ and *V*_SY_ are the volume fractions of the syntactic foam matrix and spacer yarns in the WKSF-SF sample, respectively. Regardless of the voids in WKSF-SF, the sum of *V*_NSF_ and *V*_SY_ is 1. The calculation process of *V*_SY_ is showed as follows: firstly, the cross-sectional area of the single spacer yarn was determined according to the diameter of the spacer yarn, and then, the spacer yarn’s cross-sectional area within 1cm^2^ can be determined according to the spacer yarn distribution density (shown in Table 1), and finally, by dividing this value by 1 cm^2^, we can get *V*_SY_, and *V*_NSF_ can be obtained at the same time. However, the calculation process of the *V*_NSF_ and *V*_SY_ values did not take into account the influence of voids in the composite. Therefore, in order to ensure the accuracy of the prediction results, it is necessary to introduce a new parameter, *V*_v2_, which means the extra voids’ volume fraction after the addition of the spacer yarns in the composite. By considering all the WKSF-SF samples, the value of *V*_v2_ was determined as 3% in this research. Therefore, after correcting the previously calculated *V*_NSF_ and *V*_SY_ by *V*_v2_, the final *V*_NSF_ and *V*_SY_ values are shown in Table 2.

After determining the above parameters, the compression modulus of WKSF-SF with different parameters (WKSF types, microballoons types and microballoons volume fractions) can be predicted by Equation (15).

## 4. Results and Discussions

**Figure 4:**
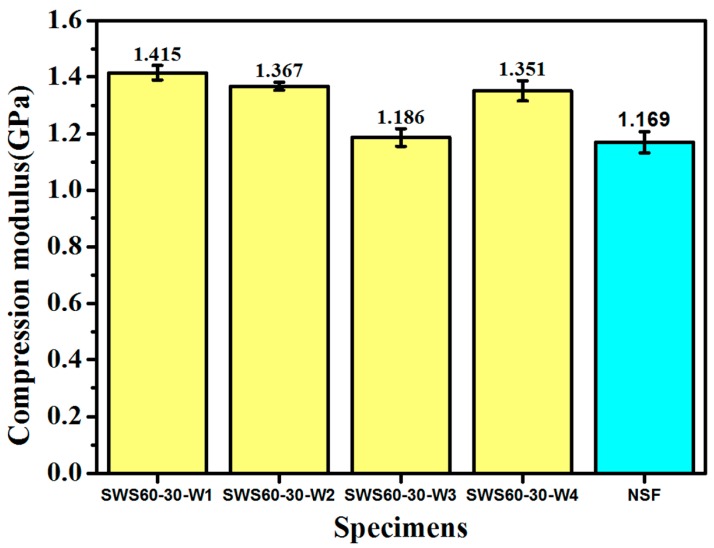
The compressive modulus of WKSF-SF and neat syntactic foam (NSF) according to the quasi-static compression test.

Figure 4 shows the compressive modulus of SF-WKSF and NSF according to the quasi-static compression test. It can be seen from the bar chart that SWS60-30-W1, SWS60-30-W2, and SWS60-30-W3 have 21%, 17% and 15% higher compressive modulus values than NSF, respectively. In addition, the compression modulus of SWS60-30-W3 is close to that of NSF. Therefore, it can be concluded that the addition of suitable WKSF produces an improvement in the compressive modulus of WKSF-SF compared with NSF.

**Figure 5:**
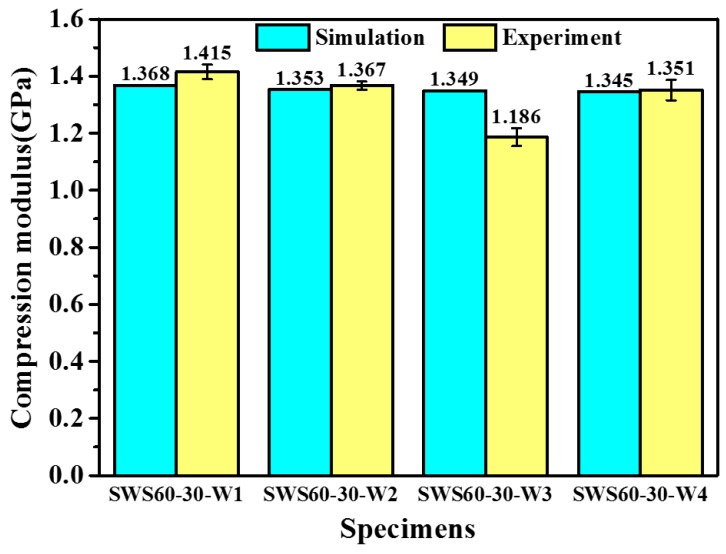
The simulation and experimental results of compression modulus for specimens reinforced with different WKSFs.

Comparisons of the experimental and simulated results of the compression modulus for WKSF-SF reinforced with different WKSFs are shown in Figure 5. It can be seen from the figure that, although a slight underestimation occurs (the difference between the theoretical values and the experimental values are less than 5%), the prediction model can accurately predict the decreasing trend of the compression modulus for SWS60-30-W1, SWS60-30-W2 and SWS60-30-W4. In addition, the compression modulus’s predicted value of SWS60-30-W3 is 14% higher than the experimental value. Meanwhile, the theoretical prediction value of SWS60-30-W3 is higher than the predicted value for SWS60-30-W4, which is inconsistent with the experiment result. The reason for this situation is that the theoretical compression modulus model in this research consists of two parts, namely the syntactic foam matrix and the spacer yarns. The syntactic foam matrixes for SWS60-30-W1, SWS60-30-W2, SWS60-30-W3 and SWS60-30-W4 are identical, that is, S60HS microballoons (the volume fraction is 30%) reinforced epoxy resin. However, these four samples have different spacer yarns volume fractions because they are embedded with different WKSFs. Since the elastic modulus of the spacer yarns is larger than that of the microballoons reinforced epoxy resin, the sample with a large *V*_SY_ (SWS60-30-W3) is predicted to have a higher compression modulus compared to the sample with lower *V*_SY_ (SWS60-30-W4) in this prediction model. On the whole, as shown in Figure 5, the prediction model can offer good prediction results for the specimens reinforced with different WKSFs.

**Figure 6:**
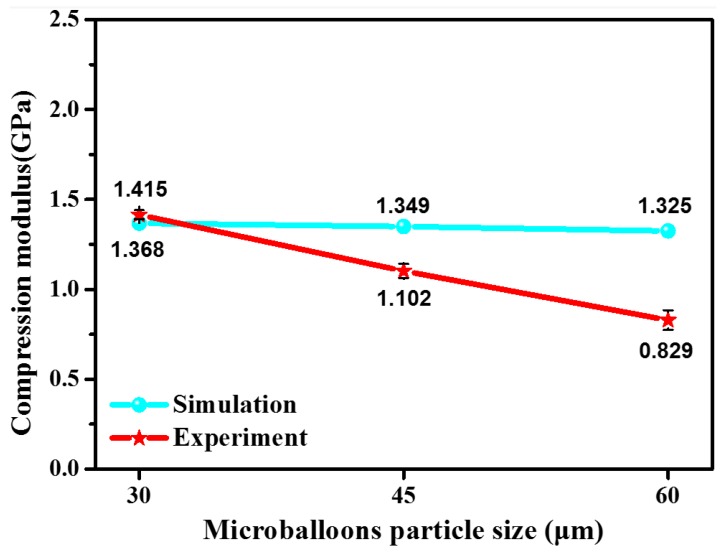
The simulation and experimental results of compression modulus for specimens with different microballoon types.

Figure 6 presents the simulated and experimental results of compression modulus for specimens with different microballoon types (characterized by microballoons’ particle sizes, which are 30, 45 and 60 μm for microballoon types S60HS, S35 and K1, corresponding to the WKSF-SF specimens SWS60-30-W1, SWS35-30-W1 and SWK1-30-W1, respectively). It can be found that the model can successfully predict the downward trend of compression modulus with the rising of microballoons particle sizes from 30 to 60 μm. It also can be found that, when the samples are reinforced with small microballoons particle size (30 and 45 μm), the results of prediction model are closer to the experimental results. However, when the microballoons’ particle size is further increased to 60 μm, the downward trend of the experiment value is obviously larger than the downward trend of the predicted value. The above situation may be due to the homogenization treatment of syntactic foam matrix. Our previous study [22] has shown that the increase in the microballoons’ particle size will reduce the interfacial shear strength between the fiber and the syntactic foam matrix, thereby affecting the mechanical properties of composite. However, the theoretical model does not involve the influence of the microballoons’ particle size on the interfacial shear strength of the composite. Therefore, when bigger microballoons (like K1 microballoons) are embedded in samples, the simulated value deviates relatively further from the experimental value.

**Figure 7:**
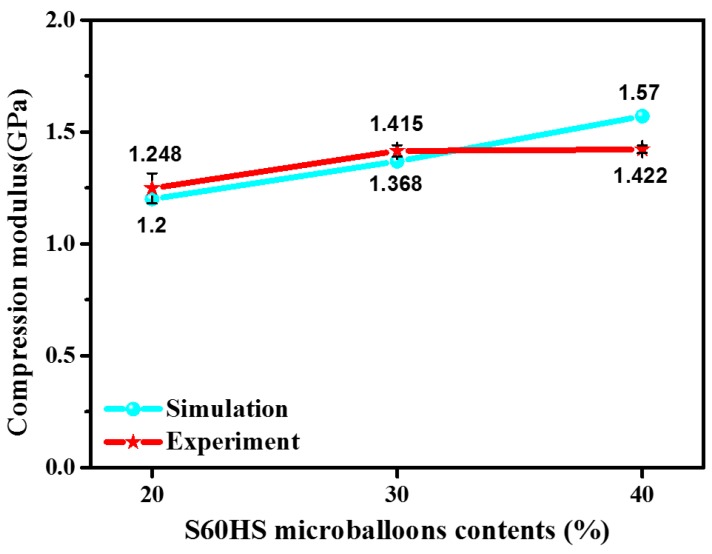
The simulation and experimental results of compression modulus for specimens with different S60HS microballoons contents.

The predicted and test results of WKSF-SF specimens with different S60HS microballoons contents are depicted in Figure 7. It is obvious that the model also accurately simulates the variation tendency of compression modulus for different samples. Meanwhile, it also can be found in Figure 7 that the simulation results of the samples with relatively lower S60HS microballoon contents are closer to the experimental result. To be specific, the experimental values of samples with 20% and 30% microballoon contents are 4% and 3% higher than the simulated values. However, when the microballoon contents of specimens increase to 40%, the simulated value is far above the experimental value (10%). This is because, in the actual situation, the addition of a large number of microballoons will increase the voids’ volume fraction *V*_v1_ in the samples, which affects the mechanical properties of the material and slows the increase trend of the compressive modulus values of materials. However, in the prediction model, the *V*_v1_ is a fixed value and determined as 0.5, which causes prediction deviations.

In general, the simulation results are in agreement with experimental results in this research, especially for the samples with a small microballoon size or moderate microballoon volume fraction. In the above situation, the compression modulus prediction results are very close to the experimental results.

## 5. Conclusions

According to the results of the quasi-static compression experiments and a comparison of the simulation and experimental values in this research for WKSF-SF, the following conclusions were established:

(1) The addition of suitable WKSF (W1, W2 and W4) produces an improvement in the compressive modulus of WKSF-SF compared with NSF;
(2) The theoretical model can effectively simulate the variation tendency of compression modulus for different WKSF-SF samples;
(3) The theoretical model is suitable for the samples with a small microballoon size or moderate microballoon volume fraction.

Based on the above conclusions, WKSF-SF can improve the absolute strength of traditional syntactic foam to a certain extent. It is a new type of syntactic foam with great potential. Meanwhile, the compression modulus meso-mechanics theoretical model built by this research can serve as a reference for practical production and further investigation of the mechanical properties of WKSF-SF, and hopefully be extended to predict the compression performance of a variety of 3D fabric-reinforced composites.

## Figures and Tables

**Table 1:** Sample specifications.

Samples	Warp-Knitted Spacer Fabric (WKSF) Type	Spacer Yarn Diameter of WKSF (mm)	Spacer Yarn Distribution Density of WKSF (number/cm^2^)	Microballoon Type	Mean Particle Size of Microballoons (μm)	Microballoon Volume Percent (%)
SWS60-30-W1	W1	0.20	57.14	S60HS	30	30
SWS60-30-W2	W2	0.20	38.97	S60HS	30	30
SWS60-30-W3	W3	0.20	30.71	S60HS	30	30
SWS60-30-W4	W4	0.16	39.52	S60HS	30	30
SWS60-20-W1	W1	0.20	57.14	S60HS	30	20
SWS60-40-W1	W1	0.20	57.14	S60HS	30	40
SWK1-30-W1	W1	0.20	57.14	K1	65	30
SWS35-30-W1	W1	0.20	57.14	S35	40	30
NSF	-	-	-	S60HS	30	30

**Table 2:** *V*_NSF_ and *V*_SY_ values.

WKSF Type	*V*_NSF_	*V*_SY_
W1	0.951	0.019
W2	0.958	0.012
W3	0.960	0.010
W4	0.962	0.008
